# A comparative evaluation of NB30, NB54 and PTC124 in translational read-through efficacy for treatment of an *USH1C* nonsense mutation

**DOI:** 10.1002/emmm.201201438

**Published:** 2012-10-02

**Authors:** Tobias Goldmann, Nora Overlack, Fabian Möller, Valery Belakhov, Michiel van Wyk, Timor Baasov, Uwe Wolfrum, Kerstin Nagel-Wolfrum

**Affiliations:** 1 Cell and Matrix Biology, Institute of Zoology, Johannes Gutenberg University of MainzMainz, Germany; 2 Edith and Joseph Fischer Enzyme Inhibitors Laboratory, Schulich Faculty of Chemistry, Technion-Israel Institute of TechnologyHaifa, Israel

**Keywords:** drug therapy, pharmacogenetics, *retinitis pigmentosa*, sensoneuronal degeneration, Usher syndrome

## Abstract

Translational read-through-inducing drugs (TRIDs) promote read-through of nonsense mutations, placing them in the spotlight of current gene-based therapeutic research. Here, we compare for the first time the relative efficacies of new-generation aminoglycosides NB30, NB54 and the chemical compound PTC124 on retinal toxicity and read-through efficacy of a nonsense mutation in the *USH1C* gene, which encodes the scaffold protein harmonin. This mutation causes the human Usher syndrome, the most common form of inherited deaf-blindness. We quantify read-through efficacy of the TRIDs in cell culture and show the restoration of harmonin function. We do not observe significant differences in the read-through efficacy of the TRIDs in retinal cultures; however, we show an excellent biocompatibility in retinal cultures with read-through *versus* toxicity evidently superior for NB54 and PTC124. In addition, *in vivo* administration of NB54 and PTC124 induced recovery of the full-length harmonin a1 with the same efficacy. The high biocompatibilities combined with the sustained read-through efficacies of these drugs emphasize the potential of NB54 and PTC124 in treating nonsense mutation-based retinal disorders.

## INTRODUCTION

**Figure 1:**
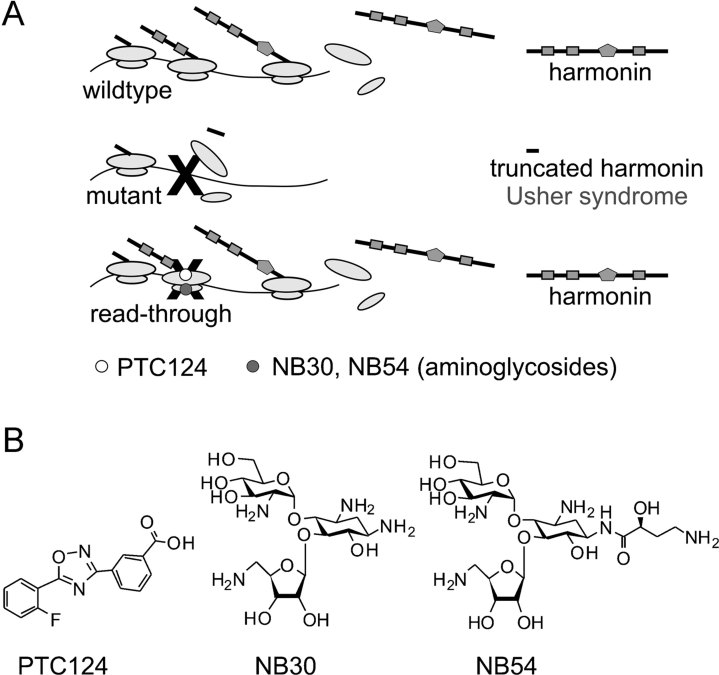
TRID induces read-through of a nonsense mutation in *USH1C* Scheme of TRID induced read-through of the p.R31X nonsense mutation. Translation of *USH1C* mRNA results in the generation of functional full-length harmonin. The p.R31X mutation introduces a premature termination codon (PTC; black X) in the mRNA, which results in truncated, nonfunctional harmonin protein leading to the human Usher syndrome. Translational read-through drugs promote the incorporation of an amino acid at the PTC of the mutant mRNA and induce generation of full-length harmonin. PTC124 (white) acts on the 60s ribosomal subunit, whereas aminoglycosides, such as NB30 and NB54 (grey) modify 40s ribosomal subunits.Chemical structures of the TRIDs: PTC124 and the new designed aminoglycosides NB30 and NB54.

In-frame nonsense mutations account for ∼12% of all hereditary disease-causing mutations (Kellermayer, 2006). A gene-based therapy that targets in-frame nonsense mutations could therefore treat a substantial proportion of patients making the approach both practical and economical. Recent studies demonstrate translational read-through (TR) to be an attractive alternative to gene therapy for in-frame nonsense mutations (Hainrichson et al, 2008; Keeling & Bedwell, 2011; Linde & Kerem, 2008; Overlack et al, 2011). During TR, the translational machinery recognizes the stop codon of the nonsense mutation as a triplet coding for an amino acid resulting in the translation of a full-length protein from mutant messenger RNA (mRNA; Fig 1A). Interestingly, various chemicals referred to as translational read-through-inducing drugs (TRIDs) are known to promote TR. Known TRIDs include clinically used aminoglycoside antibiotics like gentamicin and paromomycin, and several recently newly designed aminoglycosides like NB30 and NB54 as well as the unrelated chemical compound PTC124 (Fig 1B; Hainrichson et al, 2008; Keeling & Bedwell, 2011; Linde & Kerem, 2008).

Compared to gene addition approaches, TRIDs have significant advantages (Hainrichson et al, 2008; Linde & Kerem, 2008; Overlack et al, 2011; Zingman et al, 2007): (i) they do not act in a gene-specific manner, allowing treatment of diverse genetic conditions; (ii) the size of the causative gene and restrictions in the vector capacity are no issue, and (iii) the gene expression remains under endogenous control. Therefore, tissue and cell type specificity, timing and duration of expression as well as alternative splicing of transcripts remain largely intact. Accordingly, TRIDs make promising candidate drugs for treating multiple nonocular genetic diseases, *e.g.* cystic fibrosis (CF) and Duchenne muscular dystrophy (DMD). Current clinical trials featuring TRIDs have published encouraging results (Kerem et al, 2008; Malik et al, 2010; Politano et al, 2003; Sermet-Gaudelus et al, 2010; Wilschanski et al, 2003, 2011).

Nevertheless, the use of clinically approved aminoglycosides, *e.g.* gentamicin or paromomycin, is often associated with severe side effects including nephrotoxicity and ototoxicity, which prohibits their long-term use (Lopez-Novoa et al, 2011; Warchol, 2010). As these side effects are unrelated to the induced TR in mammalian cells, various attempts were undertaken to design more biocompatible TRIDs. One approach redesigned the chemical structures of known aminoglycosides. The designer aminoglycosides of the first generation (NB30) and the second generation (NB54) were developed by optimizing the structure-activity-toxicity relationship of the paromamine scaffold (Fig 1B; Nudelman et al, 2006, 2009). Both NB30 and NB54 were shown to induce read-through of various disease-causing nonsense mutations (Brendel et al, 2011; Goldmann et al, 2010; Lee et al, 2011; Nudelman et al, 2006, 2009; Rebibo-Sabbah et al, 2007; Rowe et al, 2011; Vecsler et al, 2011).

In addition to these aminoglycoside variants, a high-throughput drug screen aimed to identify novel TRIDs with a high biocompatibility and discovered PTC124, a chemically unrelated molecule (Fig 1B; Welch et al, 2007). The read-through efficiency of PTC124 was successfully demonstrated in animals (Du et al, 2008; Goldmann et al, 2011; Welch et al, 2007). Recent phase I and IIa/IIb clinical trials found an improvement in the symptoms of PTC124-treated CF patients with no drug-related side effects in children or adults (Hirawat et al, 2007; Kerem et al, 2008; Sermet-Gaudelus et al, 2010; Wilschanski et al, 2011). With respect to DMD, a randomized, double-blind, placebo-controlled phase IIb trial was carried out. Application of PTC124 was safe over a 48-week treatment period; however, the ambitious primary endpoint did not reach statistical significance (http://ptct.client.shareholder.com/releasedetail.cfm?ReleaseID=518941). Currently, a detailed subgroup analysis of the trial is ongoing.

In this study, we directly compare for the first time the abilities of NB30, NB54 and PTC124 to induce TR of a disease-causing nonsense mutation (p.R31X) in the human Usher syndrome type 1C (*USH1C*) gene (Zwaenepoel et al, 2001). The human Usher syndrome (USH) is the most frequent cause of combined inheritable deaf-blindness (Wolfrum, 2011; Yang, 2012). USH is a complex disease and based on heterogenic clinical courses it is divided into three clinical types (USH1-3), which are also genetically heterogeneous. The most severe form of the disease is USH1 characterized by profound prelingual hearing loss, vestibular areflexia and prepubertal onset of retinal degeneration, *retinitis pigmentosa*. The USH1 subtype comprises between 25 and 44% of all USH patients (https://grenada.lumc.nl/LOVD2/Usher_montpellier/USHbases.htlm). Within USH1, the USH1C subtype accounts for 7–14% cases (Le Quesne et al, 2012; Ouyang et al, 2003). However, due to founder effects, the incidence for USH1C is in some USH1 populations, *e.g.* the French Canadians from Quebec, up to 60% (Ebermann et al, 2007). Although none of these founder mutations of USH1C are nonsense mutations, in-frame nonsense mutations represent ∼20% of all identified different USH-causing mutations (https://grenada.lumc.nl/LOVD2/Usher_montpellier/USHbases.htlm) for which our present study serves as proof-of-principle for potential beneficial treatments of the affected patients.

The *USH1C* gene encodes the scaffold protein harmonin, which is expressed as numerous alternatively spliced isoforms (Bitner-Glindzicz et al, 2000; Verpy et al, 2000). Harmonin isoforms a and b are the key organizers in the protein networks of the interactome related to USH (Bitner-Glindzicz et al, 2000; Reiners et al, 2003; Verpy et al, 2000; Wolfrum, 2011).

While the auditory deficit in USH patients is successfully treatable with cochlear implants, so far there is no effective treatment for the retinal component of USH (Overlack et al, 2011). In the present study, we evaluated the translational read-through approach as a potential retinal treatment option by comparing NB30, NB54 and PTC124 in their translational read-through efficacy and retinal biocompatibility. We obtained different read-through efficacies of the applied TRIDs on the *USH1C* p.R31X mutation at diverse systemic levels including cell culture, retinal explants and *in vivo*. Our data conclusively highlight NB54 and PTC124 as excellent candidate drugs to treat a nonsense mutation causing USH1C.

## RESULTS

### Relative read-through activity of NB30, NB54 and PTC124 in cell culture

**Figure 2:**
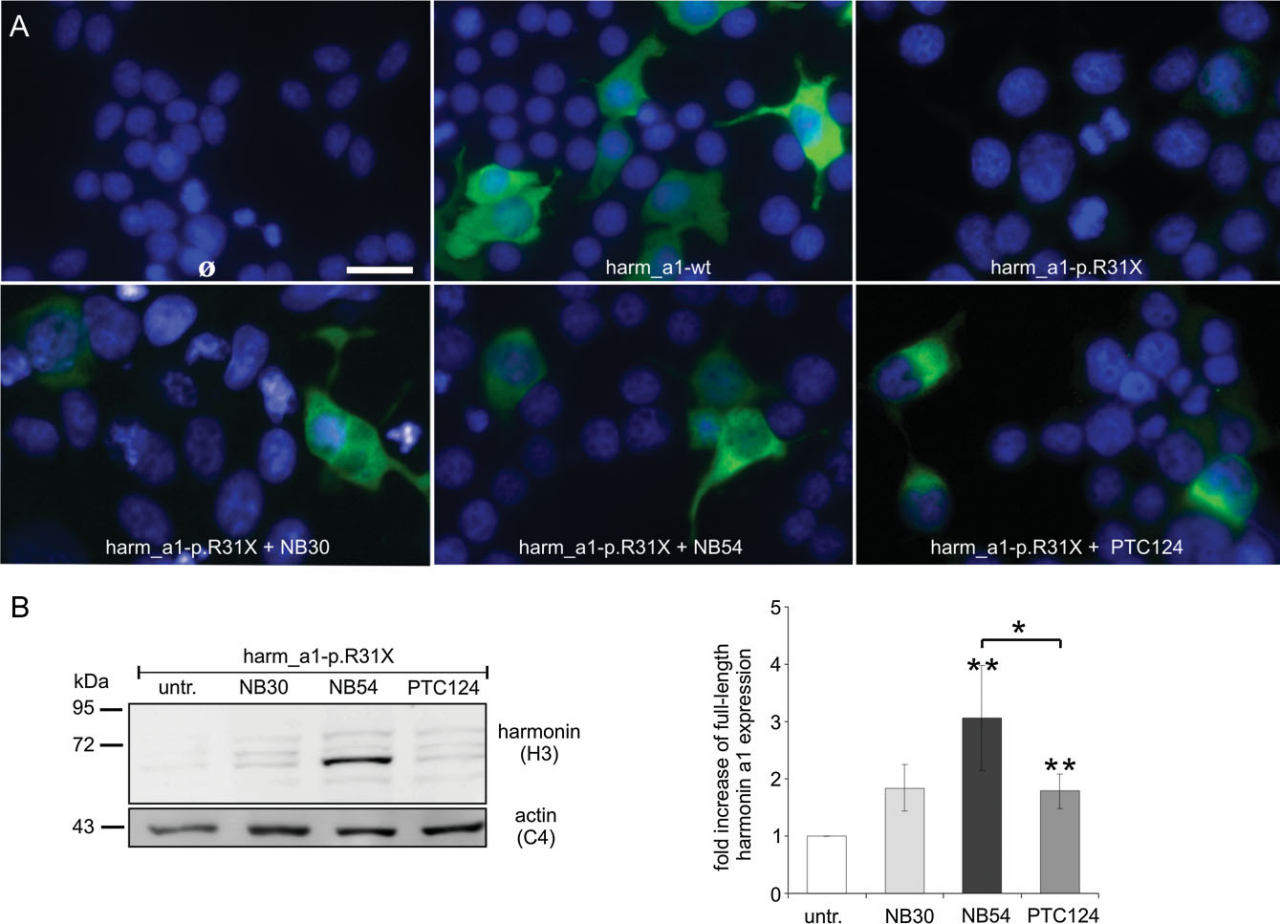
Indirect immunofluorescence and Western blot analyses of TRID-mediated read-through in HEK293T cells Read-through in transient transfected HEK293T cells analyzed by indirect immunofluorescence with anti-harmonin antibodies. Harmonin staining was detected in harm_a1-transfected cells whereas no staining was visible in untransfected cells and harm_a1-p.R31X controls. NB30 (2 mg/ml), NB54 (0.5 mg/ml) or PTC124 (10 µg/ml) treatment restored harmonin a1 (green) in p.R31X-transfected cells. Nuclear DNA was stained by DAPI (blue).Read-through in transiently transfected HEK293T cells analyzed by Western blot with anti-harmonin antibodies. Treatment with NB30, NB54 or PTC124 restored full-length harmonin a1 (∼80 kDa) in p.R31X-transfected cells. Actin staining (∼42 kDa) was used as loading control. For quantification of TRID-mediated read-through of the p.R31X mutation, the optical densities of harmonin a1 bands, stained by anti-harmonin antibodies, were measured and normalized to the appropriate loading control. The increase of read-through is shown as fold increase over untreated (untr.) cells. Quantitative data resulted from three to five independent repeats of the experiments, Error bars represent SD, *p < 0.05, **p < 0.01, scale bar 10 µm.

We compared the relative abilities of NB30, NB54 and PTC124 to induce read-through of the p.R31X mutation in the *USH1C* gene, which encodes the scaffold protein harmonin (Bitner-Glindzicz et al, 2000; Verpy et al, 2000). For this, we transfected HEK293T cells with mutated complementary DNA (cDNA) coding for the most abundant harmonin isoform, harmonin a1 and subsequently applied TRIDs at their published effective concentrations: 2 mg/ml NB30 (Goldmann et al, 2010; Rebibo-Sabbah et al, 2007), 0.5 mg/ml NB54 (Nudelman et al, 2009) or 10 µg/ml PTC124 (Goldmann et al, 2011; Welch et al, 2007). Indirect immunofluorescent labelling of harmonin revealed brightly stained cells after TRID treatment (Fig 2A). In Western blot analyses from lysates of transfected TRIDs-treated cells we detected recovered full-length harmonin a1 protein (∼80 kDa) expression (Fig 2B). We normalized the band intensity of recovered hamonin to the band intensity of actin. The quantification of band intensities showed a significant increase in harmonin expression for all TRIDs compared to untreated p.R31X-transfected cells (control). The highest relative increase in full-length harmonin following TRID treatment was 3.1-fold for NB54. The effects of NB30 and PTC124 treatment were more subtle, resulting in a 1.8-fold and a 1.7-fold increase in full-length protein, respectively (Fig 2B, Table 1).

**Table 1:** Read-through activity, restoration of protein function and biocompatibility of TRIDs

	Read-through activity			Restoration of harmonin function			TUNEL	
			
TRID	Cell culture	Retinal explants	*In vivo*	Pull-down	Active harmonin	Bundling	Mouse	Human
NB30	1.8 × 2.1%^*^ (2 mg/ml)	7.2 × (2 mg/ml)	n.a.	75% (2 mg/ml)	1.9% (2 mg/ml)	Yes 79%	1.9 × (2 mg/ml)	n.a.
NB54	3.1 × 3.7%^*^ (0.5 mg/ml)	7.9 × (0.5 mg/ml)	1.4 × (125 µg)	83% (0.5 mg/ml)	3.1% (0.5 mg/ml)	Yes 83%	1.1 × (0.5 mg/ml)	1.3 × (0.5 mg/ml)
PTC124	1.7 × 2.5%^*^ (10 µg/ml)	8.0 × (10 µg/ml)	1.4 × (2.5 µg)	80% (10 µg/ml)	2.0% (10 µg/ml)	Yes 79%	1.2 × (10 µg/ml)	1.1 × (10 µg/ml)

x-fold increase over0 its corresponding untreated control; ^*^ to its corresponding wildtype control, na, not analyzed.

To estimate the relevance of the rescued harmonin expression, we calculated the percentage of restored harmonin protein as the ratio of harmonin expression in p.R31X-transfected, TRID-treated cells to that of cells transfected with wildtype harmonin lacking the p.R31X mutation. We achieved the highest amount of recovered total harmonin expression with NB54, which yielded in a 3.7% recovery of total harmonin expression compared to 2.1 and 2.5% for NB30 and PTC124, respectively (Table 1). In summary, all TRIDs were able to rescue translational read-though of the p.R31X mutation to some degree resulting in full-length harmonin a1 expression with the highest level of read-through achieved by NB54.

Pharmacokinetic and pharmacodynamics performance indicate that NB54 and PTC124 target the translation machinery in different ways (Finkel, 2010); consequently, a synergistic effect of combined TRIDs application is reasonable. We evaluated the read-through efficiency following co-administration of NB54 and PTC124. As described above, we transfected HEK293T cells with mutated cDNA coding for harmonin a1, added NB54, PTC124 or a combination of both drugs to the cells and compared recovered full-length harmonin a1 expression in Western blot analyses (Fig S1A of Supporting Information). We normalized the band intensity of recovered harmonin to the band intensity of actin. The quantification of the normalized band intensities revealed no significant increase of harmonin expression following co-administration of NB54 or PTC124 compared to single treatments (Fig S1B of Supporting Information) indicating no synergistic effect of the co-administration of both TRIDs.

In summary, all administered TRIDs were able to rescue translational read-though of the p.R31X mutation to some degree resulting in full-length harmonin a1 expression with the highest level of read-through achieved by NB54.

### TRID-mediated read-through results in expression of functional protein

The murine *Ush1c* gene contains at least 28 coding exons, which give rise to numerous harmonin splice variants. Based on their modular structure, these splice variants have been divided into three groups (a–c; Bitner-Glindzicz et al, 2000; Reiners et al, 2006; Verpy et al, 2000). All three subclasses share a common N-terminus, which consists of two PDZ [named after its presence in the proteins postsynaptic density 95 kDa (PSD95), disc large (DLG), *Zonula Occludens* 1 (ZO-1)] domains as well as a coiled-coil domain (CC). In addition to these shared structural motifs, harmonin a isoforms contain a third PDZ domain, while harmonin b isoforms contain a second CC, a prolin–serine–threonin (PST) domain and a third PDZ domain (Reiners et al, 2003; Verpy et al, 2000). In previous publications, we have characterized harmonin as a key organizer of the USH protein interactome: the different PDZ domains are responsible for interactions with all known USH1 proteins and most USH2 proteins, including the cytoplasmic domain of the transmembrane protein USH2a (Adato et al, 2005; Reiners et al, 2005a, b; Wolfrum, 2011), while the PST domain mediates binding to actin filaments introducing actin filament bundles (Boëda et al, 2002; Wolfrum, 2011). The p.R31X mutation introduces a TGA stop codon at the N-terminus of the *Ush1c* gene leading to a truncated peptide lacking all protein–protein interacting domains described above. During TR, the introduced amino acid is not necessarily the one of the wildtype protein. Such an amino acid substitution may interfere with protein function (Keeling & Bedwell, 2011). In order to confirm the recovery of the scaffold function of restored harmonin after TRID treatment, we tested for the known interaction of the first PDZ domain of harmonin a1 with the C-terminal PDZ binding motif of the cytoplasmic domain of the transmembrane protein USH2a (Reiners et al, 2005b) as well as the ability of harmonin b to bundle actin filaments (Boëda et al, 2002).

**Figure 3:**
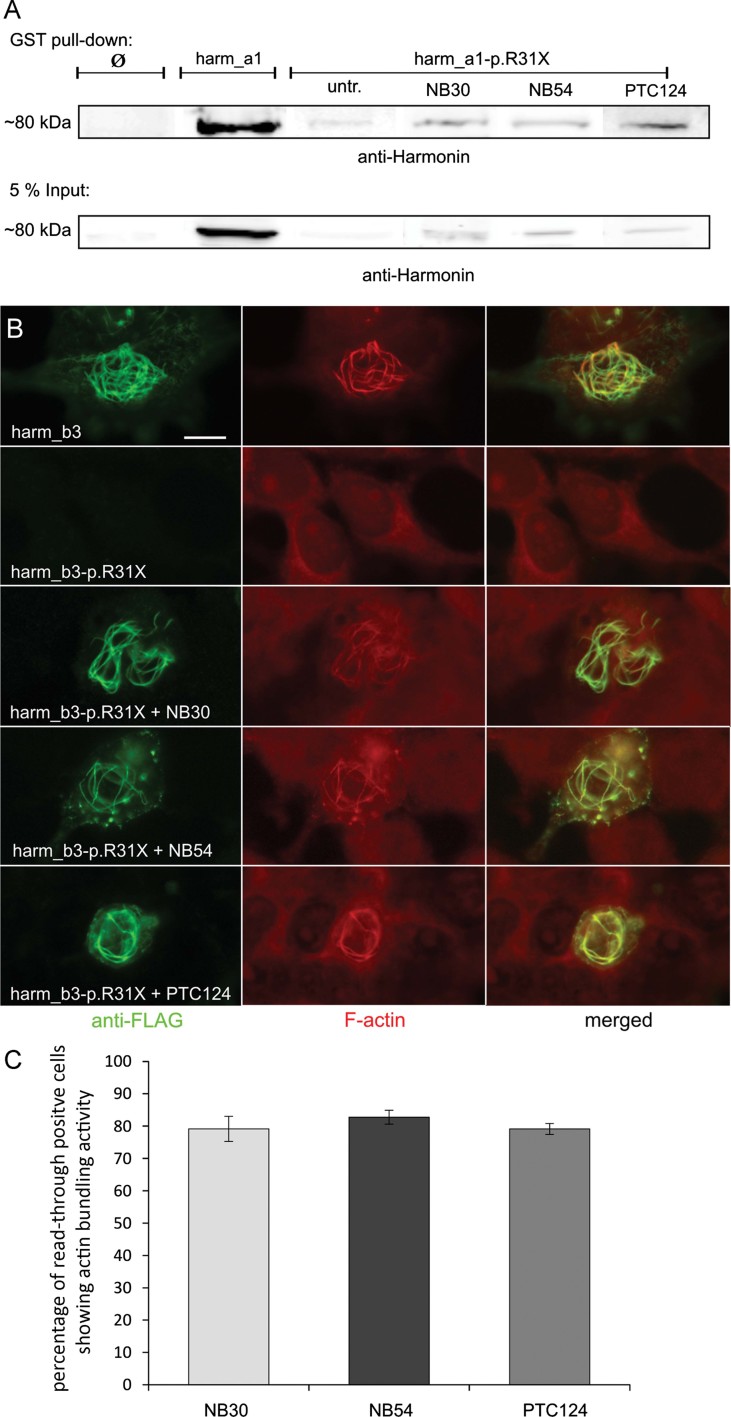
Restoration of protein function Restoration of harmonin a1 scaffold function assayed by GST pull-down. HEK293T cell lysates of harm_a1-p.R31X transfected and treated with NB30, NB54 or PTC124 were incubated with the immobilized GST-USH2a cytoplasmic (cyto) tail. Precipitated proteins were subjected to Western blot analyses using anti-harmonin antibodies. As demonstrated in harm_a1-transfected cells, TRID treatment of harm_a1-p.R31X cells restored binding of harmonin to the USH2a cyto tail.Read-through induced restoration of harmonin b3 actin filament (F-actin) bundling capacity. HEK293T cells were transfected either with FLAG-tagged wildtype harmonin b3 (harm_b3) or the p.R31X (harm_b3-p.R31X) and subsequently cultured in the absence or presence of NB30, NB54 or PTC124. Co-staining with anti-FLAG and rhodamine-phalloidin for F-actin revealed harmonin b3 expression and bundling of F-actin in wildtype and in read-through positive cells but not in controls.Percentage of read-through positive cells showing actin bundling activity. Quantitative data resulted from three independent experiments. Ø: untransfected cells, scale bar: 5 µm.

Glutathione *S*-transferase (GST) pull-down experiments using the C-terminus of USH2a as bait recovered a strong harmonin a1 band from the pooled extracts of TRID-treated cells, whereas only a very weak harmonin band was present when using extracts of p.R31X-untreated cells (Fig 3A). To determine the amount of recovered functional protein, we quantified the harmonin a1 bands in Western blot analysis of the USH2a pull-downs. For binding activity assessment the ascertained intensities of the bands were set in relation to the input of the pull-downs. Next, we compared the binding activities of TRID-recovered protein to wildtype harmonin a1. Based on these calculations the read-through of the p.R31X mutation mediated by NB54 restored about 83% of the binding activity of wildtype harmonin a1. Application of PTC124 and NB30 resulted in 80 and 75% of the binding activity of wildtype harmonin a1, respectively. Taking this result in account with the total harmonin expression in response to the TRID treatments (see Table 1, 1st lane), we achieved the highest amount of functional protein for NB54 (3.1%) compared to PTC124 (2.0%) and NB30 (1.9%; Table 1).

Double labelling with anti-FLAG antibodies and rhodamine-phalloidin revealed actin filament bundling in cells overexpressing FLAG-harmonin b3 but not FLAG-harmonin b3-p.R31X (Fig 3B). Application of either NB30, NB54 or PTC124 to the medium of FLAG-harmonin b3-p.R31X transfected cells recovered actin filament bundling indicating functionally active harmonin b3 (Fig 3B). In total, ∼80% of the TRID recovered harmonin b-expressing cells revealed an actin bundling activity (Fig 3C, Table 1), which is in line with the scaffolding function activity demonstrated above.

### Read-through of the p.R31X mutation in organotypic retina cultures

**Figure 4:**
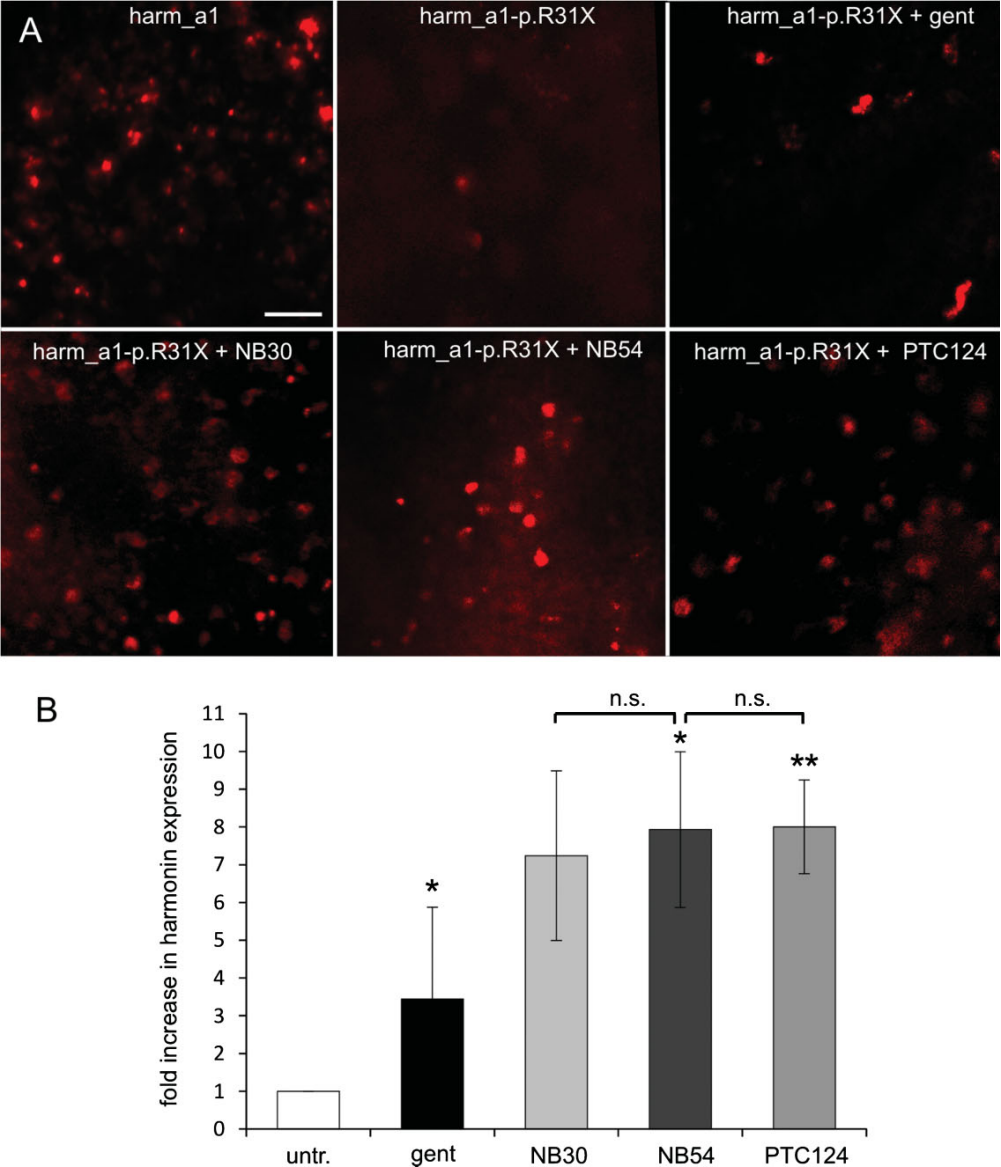
TRID-mediated read-through of the p.R31X nonsense mutation in organotypic retina cultures TRID-mediated read-through in p.R31X transfected murine retinal cultures. Retinas were transfected with harm_a1-p.R31X-mRFP, subsequently cultured in the presence of NB30, NB54 or PTC124, the harm_a1 transfection was used as control. Retinal whole mounts were analyzed by fluorescence microscopy. Expression of the red fluorescent marker indicates read-through of the p.R31X nonsense mutation.Quantification of mRFP-positive cells revealed an increase of harmonin expression after TRIDs treatment. Quantification was the result of two to four independent experiments, error bars represent SD, *p < 0.05, **p < 0.01, scale bar: 100 µm.

Harmonin isoforms are strongly expressed in photoreceptor cells where they are thought to play a crucial role in the organization and regulation of the USH protein networks (Reiners et al, 2003; Wolfrum, 2011). Accordingly, we compared the action of TRIDs on the p.R31X mutation in retinal explants. We introduced the mutated harmonin a1 C-terminally fused to a reporter coding for red-fluorescent protein (harm_a1-p.R31X-mRFP) in isolated retinas by electroporation and applied TRIDs to the medium. A small amount of spontaneous read-through was detected in control retinal explants transfected with harm_a1-p.R31X-mRFP (Fig 4A). This is in accordance with a low-level basal read-through of harm_a1-p.R31X and other nonsense mutations in transfected cultured cells (Rebibo-Sabbah et al, 2007). However, TRID treatment resulted in an increase of mRFP-expressing cells (Fig 4A). For quantification, we analyzed the number of red-fluorescent cells in seven randomly selected regions of the retinas and compared the results to untreated p.R31X-transfected control retinas (Fig 4B). NB30, NB54 and PTC124 were twice as efficient as gentamicin (3.4-fold; Fig 4B), a conventional aminoglycoside previously used in animal experiments and clinical trials (Guerin et al, 2008; Linde & Kerem, 2008; Malik et al, 2010; Moosajee et al, 2008; Wilschanski et al, 2003). PTC124 and NB54 resulted in the similar significant increase in read-through (8.0-fold and 7.9-fold, respectively) followed by a 7.2-fold increase by NB30, the first generation compound (Fig 4B, Table 1).

### High retinal biocompatibility of NB54 and PTC124

**Figure 5:**
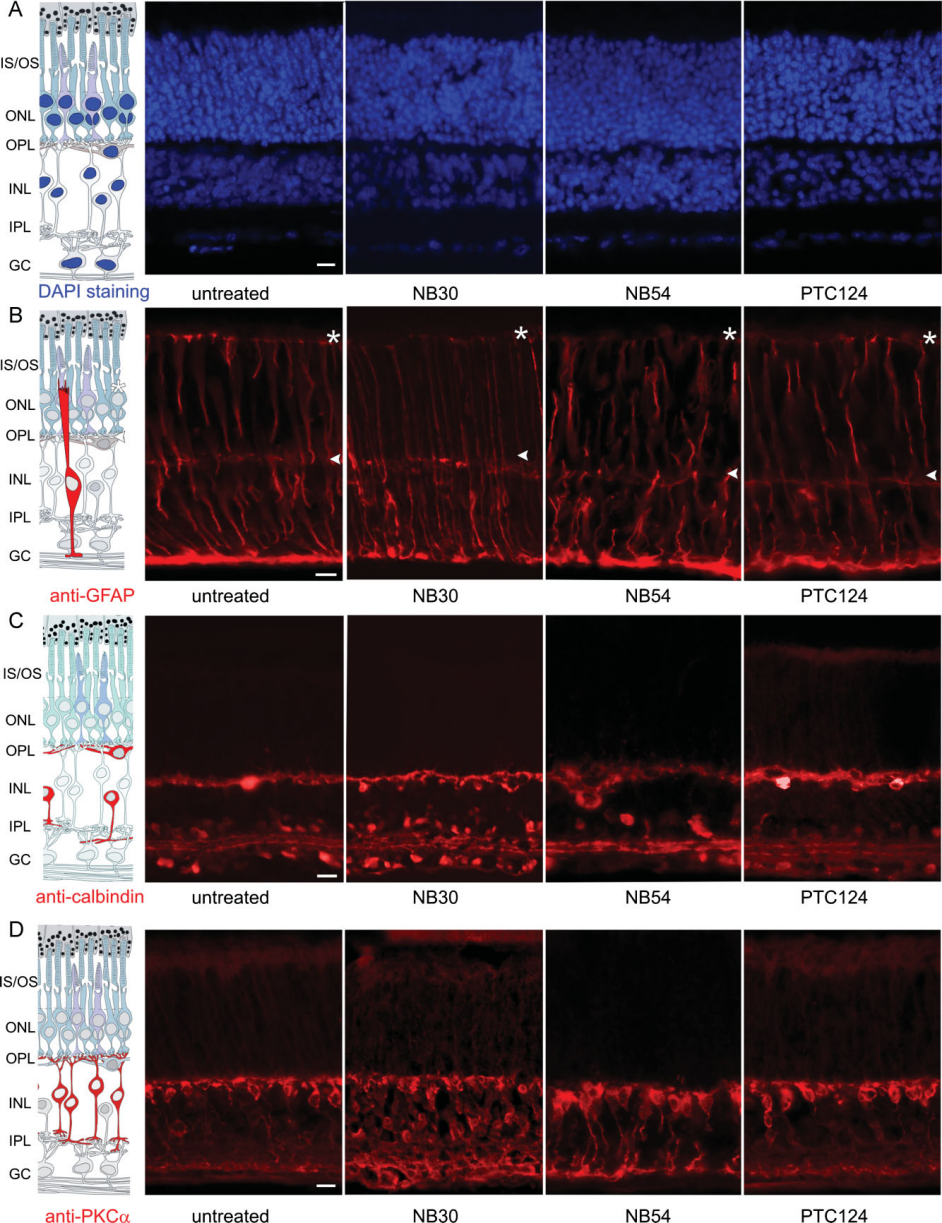
Safety profiles of TRIDs in the retina Cryosections of TRIDs-treated and untreated murine retinal cultures were analyzed for retinal integrity and cellular composition. **A.** DAPI staining of nuclear DNA visualized the well-defined layers of the neuronal retina. No alteration in the characteristic layer organization and thickness of these layers was observed after TRIDs treatment.**B–D.** Indirect immunofluorescence of retinal cell types using cell specific molecular marker antibodies: (**B**) anti-GFAP stained Müller glia cell extensions, which span from the ganglion cell layer (GC) through the inner nuclear layer (INL) and outer nuclear layer (ONL) to the *membrana externa limitans* (asterisk), (**C**) anti-calbindin stained horizontal cells and amacrine cells, (**D**) anti-PKCα labelled bipolar cells. TRIDs administration did not alter the cellular distribution of specific markers indicating no apparent effect on retinal cells and underlining the high biocompatibility. IS/OS, inner/outer segment; IPL, inner plexiform layer; OPL, outer plexiform layer, scale bars: 10 µm.

We assessed the retinal biocompatibility of NB30, NB54 and PTC124 in organotypic retina cultures, which serve as reliable tools to monitor drug effects on the retina (Goldmann et al, 2010, 2011; Maerker et al, 2008; Orisme et al, 2010; Reidel et al, 2008). To determine the biocompatibility, we monitored potential toxic effects of the TRIDs on the retina using cell-specific molecular markers to analyze retinal integrity. For this, we applied the TRIDs for 48 h to retinal explants of postnatal day 10. Subsequently, the cultures were sectioned and incubated with different molecular markers. 4′,6-Diamidino-2-phenylindole (DAPI) staining of nuclei in retinal sections revealed no difference in the structure or thickness of the well-defined layers of retinal neurons following TRID treatment (Fig 5A). In addition, we observed no apparent structural differences between TRID-treated and control retinas following indirect immunofluorescence staining against various cellular markers including glial fibrillary acidic protein (GFAP), calbindin and PKCα (Fig 5B–D).

**Figure 6:**
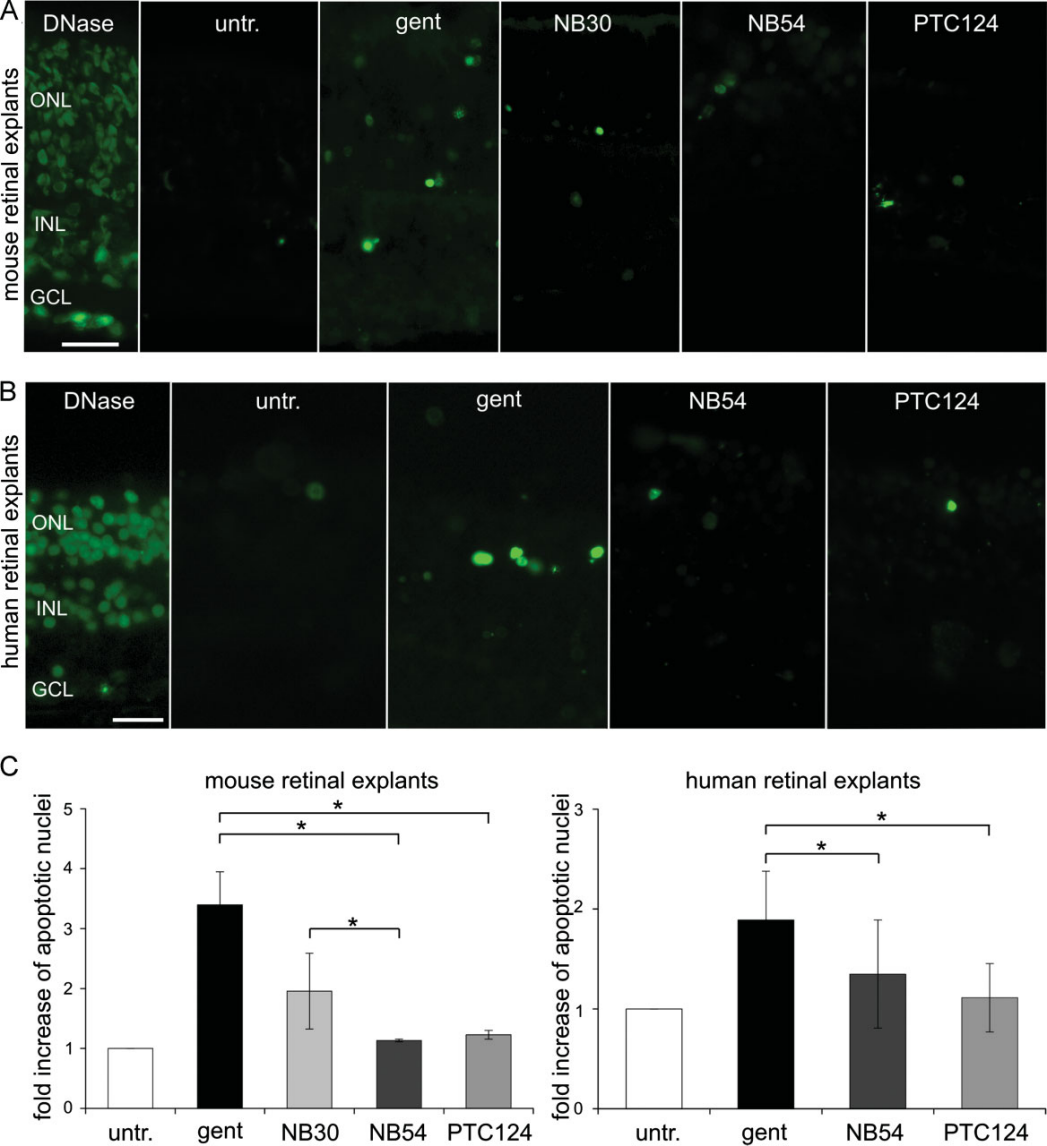
Biocompatibility of TRIDs in mouse and human organotypic retina cultures **A,B.** Fluorescence microscopy analyses of TUNEL stained sections through (**A**) mouse retina explants treated with gentamicin (gent), NB30, NB54 or PTC124 (**B**) human retinal explants treated with gent, NB54 or PTC124. DNase-treated cryosections served as positive control.**C.** Increase of apoptotic cells after TRID treatment is shown in relation to the control. Quantitative data resulted from two to four independent experiments, error bars represent SD, *p < 0.05, scale bars: 10 µm.

Furthermore, we analyzed possible TRID-induced cell death after drug treatment in organotypic retina cultures using TdT-mediated dUTP nick end labelling (assay) (TUNEL) assays. In this set of experiments, we included gentamicin as a clinically used aminoglycoside as control (Goldmann et al, 2010, 2011). In untreated control retinas, a low level of TUNEL-positive nuclei was present whereas in gentamicin- or NB30-treated cultures an increase of stained nuclei was visible (Fig 6A). In contrast, no obvious toxic effect was detectable in response to NB54 or PTC124 treatment (Fig 6A). The quantification of TUNEL-positive nuclei revealed an increase in the number of dead cells in murine retinal cultures treated with gentamicin or NB30 compared to control cultures (Fig 6A and C, Table 1). However, no significant alteration was observed after NB54 or PTC124 treatment compared to the control. The direct comparison between all TRIDs revealed a significantly lower number of TUNEL-positive cells of PTC124- or NB54-treated cultures compared to gentamicin (Fig 6A and C). We observed no significant difference between the clinically approved PTC124 and the designer aminoglycoside of the 2nd generation, NB54. Since NB54 and PTC124 revealed a significantly better biocompatibility than NB30, we focused on these two TRIDs and excluded NB30 from the following experiments.

Next, we tested the biocompatibility of TRIDs in human retinal explants cultured from human donor eyes post-mortem. Gentamicin treatment resulted in an increase of cell death, whereas no increase in apoptotic cells was detected in NB54- or PTC124-treated explants, which is in line with the analyses in murine retina cultures. Quantification of data revealed that NB54 or PTC124 application to human retinal cultures resulted in no significant difference in the number of apoptotic cells compared to untreated control retinas (Fig 6B and C, Table 1).

### NB54 and PTC124 induce read-through in the retina *in vivo*

**Figure 7:**
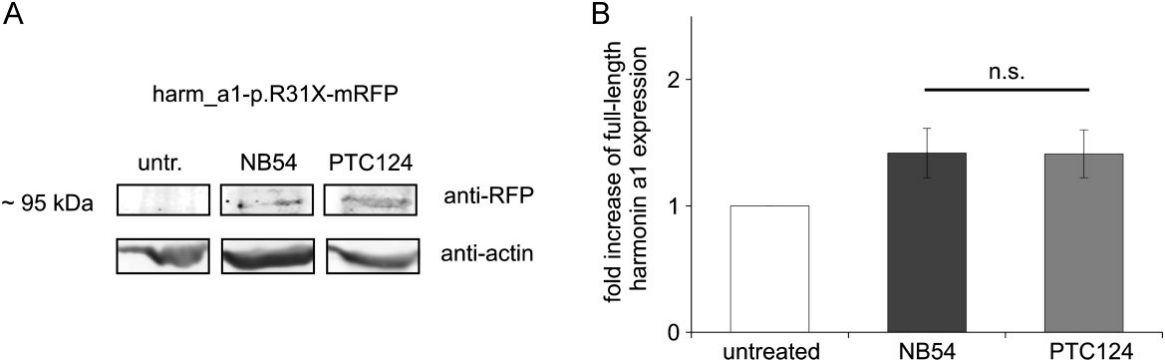
Western blot analyses of read-through in the retinas *in vivo* Western blot analyses of NB54 or PTC124 mediated read-through in the retina of harm_a1-p.R31X-RFP electroporated mice. Subretinal injections of PTC124 or NB54 restored full-length harmonin a1-mRFP expression. Actin-staining (∼42 kDa) was used as loading control.For quantification of read-through of the p.R31X mutation, the optical densities of harmonin a1-mRFP, stained by anti-RFP antibodies, were ascertained and normalized to the appropriate loading control. The increase of read-through is shown as fold increase over control mice. Quantitative data resulted from two to three independent experiments; error bars represent SD, n.s. not significant.

The read-through ability of TRIDs was examined *in vivo* in the retinas of mice. Since no mouse model for the p.R31X mutation exists, we used the *in vivo* electroporation technique to transfer harm_a1-p.R31X-mRFP reporter constructs into the retina of newborn mouse pups (Goldmann et al, 2011; Matsuda & Cepko, 2004). Six weeks after the electroporation, NB54 or PTC124 were injected subretinally into the electroporated mouse eye and read-through was assessed by Western blot analyses (Fig. 7). Applying anti-RFP antibodies, we did not observe protein expression in harm_a1-p.R31X-mRFP control retinas, however, in NB54- and PTC124-injected retinas, we detected full-length harmonin a1 (Fig 7A). Quantification of the read-through levels revealed a 1.4-fold increase in both PTC124- and NB54-treated retinas with no significant difference (Fig 7B).

## DISCUSSION

TR is an attractive alternative gene-based therapy for in-frame nonsense mutations, which account for at least 12% of all hereditary disease-causing mutations (Hainrichson et al, 2008; Kellermayer, 2006; Keeling & Bedwell, 2011; Linde & Kerem, 2008; Overlack et al, 2011). In the present study, we compared the relative abilities of two different designer aminoglycosides, namely NB30 and NB54, as well as the chemical compound PTC124 to induce read-through of an in-frame nonsense mutation for the first time. All individually administered TRIDs were able to rescue translational read-though of the p.R31X mutation in the human *USH1C* gene (Zwaenepoel et al, 2001) to some degree resulting in full-length harmonin a1 with the highest level of read-through achieved by NB54.

In the human retina, *USH1C* isoforms of all three harmonin subclasses are expressed (Nagel-Wolfrum et al, 2011). However, so far it is unknown which isoform(s) is/are essential for retinal function, hampering gene addition approaches and demonstrating the importance to develop alternative strategies for patients with USH1C. Here, we demonstrate TR as a promising therapy strategy for USH1C. We showed that NB30, NB54 and PTC124 were able to induce TR of the p.R31X-nonsense mutation of *Ush1c* and that the recovered full-length harmonin isoforms, which are thought to be essential for retinal function, are functional (Reiners et al, 2003, 2005b). TR of an UGA stop codon typically introduces one of three different amino acids: arginine, cysteine or tryptophan (Feng et al, 1990). Therefore, TR of the p.R31X mutation may result in full-length harmonin containing cysteine or tryptophan instead of an arginine amino acid at the mutation site. However, present data demonstrate that more than 80% of the recovered harmonin is functional. Thus, protein function is reestablished despite of a possible incorrect amino acid incorporated in the recovered harmonin. Interestingly, incorporation of faulty amino acids, as caused by missense mutations in *USH1C*, results only in isolated deafness but not in retinal degeneration (Ahmed et al, 2002; Ouyang et al, 2002; Reiners et al, 2006; Yan & Liu, 2010). Based on the phenotype-genotype correlation, any TRID-mediated read-through of the p.R31X mutation in *USH1C* may be sufficient for the restoration of retinal function. Although the critical level of harmonin protein necessary to prevent retinal degeneration in USH1C patients is unknown, it is conceivable that even a relatively small recovery could slow down the progression of the disease as discussed for other recessive disorders (Kellermayer, 2006; Maire, 2001).

Present data show that PTC124 and NB54 induce read-through of the USH-causing nonsense mutation *ex vivo* and *in vivo* in the retina with nearly the same efficiency. Recently, systemic application of gentamicin showed a slight improvement of retinal function in a rodent model of retinal degeneration (Guerin et al, 2008). This previously published data with gentamicin in conjunction with the present significant elevated read-through efficiency of NB54 or PTC124 compared to that of gentamicin, suggests that these two TRIDs should induce the recovery of sufficient amounts of functional harmonin protein to combat or at least slow down the retinal degeneration in USH1 patients. Moreover a combined treatment of TRIDs with neurotropic factors may have an even higher capacity to treat nonsense mutation-based retinal diseases as indicated by Gregory-Evans et al (2011). Finally, in further investigations, the co-administration of poly-l-aspartic acid (PAA), which has been shown to enhance aminoglycoside-induced read-through and decrease aminoglycoside-induced toxicity, may provide an additional approach to enhance the efficacy of aminoglycoside variants NB30 and NB54 for the treatment of USH1 (Swan et al, 1991).

The read-through therapy might have an inherent problem due to potential off-target effects, *e.g.* on normal protein translation processes or the reactivation of pseudogenes, inducing side effects. Thus, the biocompatibility of TRIDs in different tissues and in the organism is an important concern (Linde & Kerem, 2008). Systemic application of clinically applied aminoglycosides, *e.g.* gentamicin, is ototoxic and/or nephrotoxic, which prohibits its long-term clinical use (Lopez-Novoa et al, 2011; Warchol, 2010). The toxicity of aminoglycosides is based on a combination of different factors, *e.g.* the formation of free radicals or binding to phospholipids (Hainrichson et al, 2008; Warchol, 2010). Additionally, cytotoxicity of aminoglycosides can result from binding and interfering with the mitochondrial rRNA, which is closely related to the bacterial rRNA (Hainrichson et al, 2008). Since this aminoglycoside toxicity is not caused by stop codon suppression during cellular protein translation, it is feasible that structural elements within aminoglycosides that induce toxicity can be separated from those that induce PTC suppression. The redesigned aminoglycosides NB30 and NB54 have a validated improved biocompatibility in cell culture and systemically in animals (Brendel et al, 2011; Lee et al, 2011; Nudelman et al, 2006, 2009; Rebibo-Sabbah et al, 2007). PTC124 has been shown to be well tolerated in animals and man (Hirawat et al, 2007; Welch et al, 2007). Here, we compared the safety profiles of NB30, NB54 and PTC124 in the retina. Since we did not detect any alterations in the retinal structure or in specific cells types following the application of all three TRIDs, we exclude any severe toxic affect of any of the analyzed TRIDs in the retina. However, our TUNEL assays revealed significant better biocompatibility of NB54 and PTC124 compared to NB30 in the murine retina. The excellent retinal biocompatibility of the aminoglycoside NB54 and PTC124 was further confirmed in a human donor retina.

These results further support the previously reported high tolerance of the newly designed aminoglycoside NB54 and PTC124 in cells, animals and humans (Brendel et al, 2011; Hirawat et al, 2007; Lee et al, 2011; Nudelman et al, 2009). All in all, we did not find any indication for harmfully prolonged protein translation or reactivation of evolutionary turned-off pseudogenes induced by TRIDs-mediated read-through of normal termination codons. Thereby, our data further support the hypothesis that normal and premature termination differ mechanistically (Welch et al, 2007). Due to *in vivo* efficacy and the observed biocompatibility, NB54 or PTC124 may be employed for use in younger patients to delay the onset of retinal degeneration in USH1C patients.

In our present study we have compared for the first time the relative efficacy of the previously introduced TRIDs, the designer aminoglycosides NB30 and NB54 along with the PTC124 compound, for read-through therapy of an *USH1C* nonsense mutation. The observed data in translational read-through, restoration of protein function and retinal biocompatibility clearly favor the 2nd generation designer aminoglycosides NB54 and PTC124 compared to the clinically applied aminoglycoside gentamicin and the first generation designer aminoglycoside NB30. Both NB54 and PTC124 induced read-through of the USH causing p.R31X nonsense mutation *in vivo* with the same efficiency. Our data underline the effectiveness and importance of the continuous improvement of TRIDs as a potential treatment of genetic diseases caused by nonsense mutations. In this regard, NB54 provides a promising scaffold for the development of new derivatives of aminoglycosides with improved biocompatibility and greater read-through efficacy of USH nonsense mutations as well as other diseases caused by nonsense mutations (Kandasamy et al, 2011; Nudelman et al, 2010). Here, we demonstrate the potential of NB54 and PTC124 as a read-through therapy to combat nonsense mutation-based retinal disorders and other groups of genetic disorders with limited or no current therapeutic options and raises hope for future clinical trials.

## MATERIALS AND METHODS

### Human material

Human donor eyes were obtained from the Department of Ophthalmology, University Medical Center of the Johannes Gutenberg University Mainz, Germany. All donors gave their written informed consent. All protocols for the donation were approved by the Institutional Ethics Committee of the University Medical Center of the Johannes Gutenberg University Mainz in accordance with the ethical standards laid down in the Declaration of Helsinki (http://www.wma.net/en/30publications/10policies/b3/).

### Animals

C57BL/6J mice were maintained on 12/12 h light (200 lux)/dark cycle, with food and water *ad libitum*. ARVO statements and institutional guidelines for animal care were followed. The animal experiments were approved the State Authority of Rhineland-Palatinate (LUA Koblenz, Germany).

### TRIDs

NB30 and NB54 were synthesized and characterized as previously reported (Nudelman et al, 2006, 2009). PTC124, kindly provided by Dr. Vladimir Maslenko (Exchemistry, Moscow, Russia), was dissolved in dimethyl sulphoxide (DMSO) (Sigma–Aldrich, Deisenhofen, Germany). Gentamicin was purchased from Sigma–Aldrich. As controls for NB30 and NB54 H_2_O, for PTC124 DMSO was used in equivalent amounts. For evaluating the potential of a synergistic activity of NB54 and PTC124; 0.5 mg/ml NB54 and 5 µg PTC124, in single or in co-administration were used.

### Antibodies and dyes

Affinity-purified polyclonal rabbit antibodies against harmonin (H3) were used as previously characterized (Reiners et al, 2003). Polyclonal rabbit antibodies against the GFAP were obtained from DAKO (Glostrup, Denmark). Antibodies against calbindin (Swant, Bellinzona, Switzerland) and PKCα (Sigma–Aldrich) were previously characterized (Haverkamp & Wassle, 2000). Monoclonal mouse antibodies to actin (clone C4) and to FLAG were purchased from Seven Hill Bioreagents (Cincinnati, OH, USA) and Sigma–Aldrich, respectively. Monoclonal rat antibodies against red fluorescent protein (RFP) were obtained from Chromotek (Martinsried, Germany). Actin filaments were visualized by rhodamine-phalloidin (Sigma–Aldrich). Secondary antibodies conjugated to Alexa 488 were obtained from Molecular Probes (Leiden, Netherlands). DAPI was obtained from Sigma–Aldrich.

### Plasmid cloning

The murine cDNA of harmonin a1 was amplified and inserted into the pCS2 + MT vector, encoding myc-tags, or the pTER-mRFP vector, encoding monomeric red-fluorescent protein (mRFP; Goldmann et al, 2010, 2011). cDNA of human harmonin b3 was subcloned into the pDest/C-SF-TAP vector, encoding a S-FLAG (Gloeckner et al, 2007). All introduced tags were located at the C-terminus of the harmonin isoforms. The p.R31X mutation was generated in all plasmids by the QuickChange Lightning Site-Directed Mutagenesis Kit (Stratgene, La Jolla, CA).

**The paper explained**

PROBLEM:

Gene addition by viral vectors is traded as a promising strategy for the treatment of hereditary disorders, including retinal diseases. However, many genes are frequently alternatively spliced and their coding sequences are too large for any available viral delivery system. On the other hand, molecular diagnostic allows selection of patients for application of mutation-specific therapies. Such a personalized therapy is the read-through strategy for nonsense mutations promoted by translational read-through inducing drugs (TRIDs). The most promising TRIDs are new-generation aminoglycosides and the chemical compound PTC124. So far, no direct comparison of read-through efficiency and biocompatibility of the two classes of TRIDs exist.

RESULTS:

Here, we compare for the first time the relative efficacies of two new-generation aminoglycosides (NB30, NB54) and PTC124 in recovering the pathophysiology of a nonsense mutation in the *USH1C* gene, which encodes the scaffold protein harmonin. This mutation causes the human USH, the most common form of inherited deaf-blindness. We quantify the read-through efficacy of three different TRIDs in cell as well as retinal cultures and demonstrate the restoration of harmonin protein function. Although we do not observe significant differences in the read-through efficacy of the three TRIDs, we demonstrate an excellent biocompatibility of NB54 and PTC124 in retinal cultures with a superior read-through *versus* toxicity ratio. In addition, *in vivo* administration of NB54 and PTC124 induced recovery of the full-length harmonin a1 with the same efficacy.

IMPACT:

For the first time, we directly compare the efficacy of translational read-through inducing drugs (TRIDs) as an attractive alternative to gene addition for in-frame nonsense mutations. The high biocompatibilities combined with the good read-through efficacies of these drugs emphasize the potential of NB54 and PTC124 in treating nonsense mutation-based retinal disorders. Furthermore, our data highlight the potential of redesigning aminoglycosides to TRIDs with even better read-through activity and reduced toxicity.

### Cell culture and protein–protein interaction analyses

HEK293T cells were grown in Dulbecco's Modified Eagle Medium with GlutaMax™ supplemented with 10% foetal calf serum (Invitrogen, Karlsruhe, Germany) and 1% penicillin/streptomycin (Invitrogen) at 37°C and 5% CO_2_. Transfections were performed, with Lipofectamine™ LTX and PLUS™ reagent (Invitrogen) according to the manufacturer's protocol. Immunofluorescence analyses of HEK293T cells were performed as previously described (Overlack et al, 2008). Actin filament bundling of harmonin b3, was analyzed with rhodamine-phalloidin. Harvesting of cells and Western blot analyses were performed as previously described (Maerker et al, 2008; Nagel-Wolfrum et al, 2004). Western blots were analyzed using Odyssey infra-red imaging system (LI-COR Biosciences, Lincoln, NE, USA). For the quantification, the relative band intensities of harmonin were normalized on the relative band intensities actin loading control. The obtained number for each TRID was set in relation to its appropriate p.R31X control. GST pull-downs were performed as previously described (Maerker et al, 2008; Reiners et al, 2005b). The optical density of the TRID induced harmonin band was ascertained and normalized to 5% of the input. The percentage of functional protein was calculated as the ratio of harmonin a1 transfected cells to p.R31X transfected TRID treated cells.

### Retina culture and electroporation

Murine and human retinas were prepared as previously described (Goldmann et al, 2011; Reidel et al, 2006). For biocompatibility, PN10 retinas were incubated for 2 days. TUNEL (TUNEL, TdT-mediated X-dUTP nick end labelling) labelling was performed according to the manufacture's protocol of ‘In Situ Cell Death Detection Kit’ (Boehringer Mannheim, Germany). Retinal explants of PN6 were electroporated as previously described (Goldmann et al, 2011; Matsuda & Cepko, 2004). Retinas were cultured for 24 h before adding the pharmacological compounds in fresh medium for additional 72 h.

### *In vivo* electroporation and subretinal TRID injection

Plasmid DNA was transferred by electroporation into retinas of postnatal day 0 mouse pups as previously described (Matsuda & Cepko, 2004). After 6 weeks, mice were anesthetized and 1 µl of NB54 (125 µg/µl), PTC124 (2.5 µg/µl) or an appropriate control was injected subretinally. Three days after the injection, animals were sacrificed and retinas were removed, homogenized and processed for Western blot analyses as described (Goldmann et al, 2011; Reiners et al, 2003).

### Microscopy

Light microscopy analyses of immunofluorescence and epifluorescence samples were performed with a Leica DM 6000 B (Leica microsystems, Bensheim, Germany). Obtained images were processed with Adobe Photoshop CS (Adobe Systems, San Jose, CA, USA).

### Statistical analysis

One-way *t*-test analysis was applied for statistical significance.

## Supplementary material

Detailed facts of importance to specialist readers are published as ”Supporting Information”. Such documents are peer-reviewed, but not copy-edited or typeset. They are made available as submitted by the authors.
