# Supplementary material for: A comparative evaluation of NB30, NB54 and PTC124 in translational read-through efficacy for treatment of an USH1C nonsense mutation
Source: EMBO Mol Med. 2012 Oct 2;4(11):1186–99. doi: 10.1002/emmm.201201438 (PMC3494875; doi:10.1002/emmm.201201438)
Supplement: Supplementary file 1 [file emmm0004-1186-SD1.pdf]

EMBO
Molecular Medicine

Manuscript EMM-2012-01438

A comparative evaluation of NB30, NB54 and PTC124 in translational read-through efficacy for treatment of an USH1C nonsense mutation

Tobias Goldmann, Nora Overlack, Fabian Möller, Valery Belakhov, Michiel van Wyk, Timor Baasov, Uwe Wolfrum, and Kerstin Nagel-Wolfrum

Corresponding author: Kerstin Nagel-Wolfrum, Johannes Gutenberg University Mainz

Review timeline:

Submission date:	11 April 2012
Editorial Decision:	15 May 2012
Revision received:	15 June 2012
Editorial Decision:	16 July 2012
Revision received:	17 August 2012
Accepted:	22 August 2012

Transaction Report:

(Note: With the exception of the correction of typographical or spelling errors that could be a source of ambiguity, letters and reports are not edited. The original formatting of letters and referee reports may not be reflected in this compilation.)

1st Editorial Decision 15 May 2012

Thank you for the submission of your manuscript to EMBO Molecular Medicine. We have now heard back from the three referees whom we asked to evaluate your manuscript. Although the referees find the study to be of potential interest, they also raise a number of concerns that should be convincingly addressed in a major revision of the present manuscript.

As you will see from the reports below, they all find the topic of your manuscript important and potentially suitable for publication. However, they feel that the data need to be strengthened in places and make constructive suggestions for that. Should you be able to address these criticisms in full, we would be willing to consider a revised manuscript.

Revised manuscripts should be submitted within three months of a request for revision; they will otherwise be treated as new submissions, except under exceptional circumstances in which a short extension is obtained from the editor. Also, the length of the revised manuscript may not exceed 60,000 characters (including spaces) and, including figures, the paper must ultimately fit onto optimally ten pages of the journal. You may consider including any peripheral data (but not methods in their entirety) in the form of Supplementary information.

I look forward to seeing a revised form of your manuscript as soon as possible.

Yours sincerely,

Editor
EMBO Molecular Medicine

***** Reviewer's comments *****

Referee #1:

Goldmann et al. have carried out comparison of different translational readthrough drugs, including PTC124 and designer aminoglycosides, using an USH1C nonsense allele as a model. This is a very comprehensive study that substantially furthers our insight into this therapeutic strategy. Most potential downsides of this approach have been addressed experimentally and are being adequately discussed. I have some minor comments which are listed below:

The functional readouts are specific for USH1C function, and although it may be anticipated that the results would be similar for other USH gene nonsense mutations, the results of this study are encouraging especially for USH1C. Therefore, some numbers should be given on the relative contribution of USH1C nonsense mutations to the USH1 subtype, especially in populations where USH1C is more prevalent because of founder effects.

How about the relative frequency nonsense mutations in other USH genes? There is a frequent PCDH15 nonsense mutation in Ashkenazi jews whose carriers would benefit from TRID-based therapies. Are there more recurrent nonsense mutations in USH genes?

The study takes most concerns about TRIDs into account, using a variety of experiments. In particular, the assessment of the functional properties of rescued harmonin is important to (largely) rule out adversive effects of the random residue that is inserted at the position of the premature stop codon. One concern, however, is the unwanted readthrough in pseudogenes that may be reactivated and potentially cause side effects. A possible way to address this would be an assay that looks at the readthrough of genes that have been inactivated very recently in human evolution and that are still largely intact apart from recently gained nonsense mutations. I think it would be beyond the scope of this study to add such experiments for a revision, but I think that this issue needs to be discussed at least.

Would the co-administration of NB54 and PTC124 be benefitial in terms of enhanced recovery, as an alternative or in addition to co-administered PAA (which has been shown to be low)? A very good point in this study is that besides pure quantification of recovered FL protein, the functional recovery has been assayed.

Referee #2:

This manuscript reports a comparison of 3 translational read-through inducing drugs (TRIDs) on retinal toxicity and read-through efficacy of a nonsense mutation in the USH1C gene that is responsible for Usher syndrome. Although 2 of the TRIDs have been compared to other aminoglycosides previously by the same authors, a relative comparison of these 3 has not yet been reported. Furthermore, currently there is no treatment option for the retinal degeneration associated with Usher, making the continued evaluation of new therapeutic strategies of high clinical importance.
The authors report that all 3 TRIDs have similar read-through efficacy and restoration of harmonin function in cell culture, while only 2 showed low toxicity. These 2 were then evaluated in vivo. Since no mouse model of USH1C.R31X exists, in vivo electroporation with reporter constructs was used. The authors report sustained read-through efficacies and high biocompatibilities of these TRIDs in vivo.
Overall, the results are supported by solid and well-controlled experiments. However the authors are a bit overenthusiastic in describing some of the results.
1) The authors describe the read-through efficacy in vivo as being sustained, however only a single evaluation of harmonin a1 was examined 72 hours post treatment. Later time points would need to be evaluated to support concluding a sustained effect, otherwise the effect should be described as transient.

2) The authors state that all three harmonin subclasses are expressed in the retina, yet only one subclass, a1, was evaluated in vivo. An analysis of harmonin b would greatly strengthen the conclusion of in vivo efficacy.

Referee #3:

It is more valuable if the author could include the comparison between NB30, NB54, PTC124 and NB84.
In page#4 on the line#18, The author should describe that PTC proceeded to phase#3 for clinical trial for DMD patients although it has been suspended.
The data shown in figs. 2B, 4B and 7A, the author should indicate the level of wild type animals. Otherwise, readers can not understand the significance of the data cleary.
In page#16, it may be possible that the author used not one-way t-test but one-way ANOVA.

1st Revision - Authors' Response 15 June 2012

On behalf of my co-authors and myself, I like to thank you and the reviewers for consideration and for the evaluation of our manuscript and for the encouraging comments and helpful suggestions. The responses to the individual points are stated below. Paragraphs from the manuscript are in italics, actual changes we made to the manuscript are underlined. Furthermore, we added a version of our revised manuscript containing yellow marks of all changes we have made.

Response to Referee #1:

Reviewer 1 highlights that our study is very comprehensive and that it substantially furthers the insights into translational read-through therapeutic strategy. Furthermore she/he attests that we have addressed experimentally and have adequately discussed most potential downsides of this approach.

A point-to-point discussion follows:

Comment 1: The functional readouts are specific for USH1C function, and although it may be anticipated that the results would be similar for other USH gene nonsense mutations, the results of this study are encouraging especially for USH1C. Therefore, some numbers should be given on the relative contribution of USH1C nonsense mutations to the USH1 subtype, especially in populations where USH1C is more prevalent because of founder effects.

Response to comment 1: Following reviewer´s comment we included more information on the relative contribution of nonsense mutations to the USH subtype and stress the high prevalence of USH1C in founder populations. For this we include on page 5 of the Introduction following sentences:

> *"The USH1 subtype comprises between 25-44% of all USH patients (https://grenada.lumc.nl/LOVD2/Usher_montpellier/USHbases.htlm). Within USH1 the USH1C subtype account for 7-14% cases {Ouyang 2005; Stabej 2012}. However, due to founder effects the incidence for USH1C is in some populations,*
>
> *e.g. the French Canadians from Quebec, up to 60% {Ebermann 2007}. Although none of these founder mutations of USH1C are nonsense mutations, in-frame nonsense mutations represent ~ 20% of all identified different USH causing mutations (https://grenada.lumc.nl/LOVD2/Usher_montpellier/USHbases.htlm) for which our present study serves as proof of principle for potential beneficial treatments of the effected patients."*

Comment 2) How about the relative frequency nonsense mutations in other USH genes? There is a frequent PCDH15 nonsense mutation in Ashkenazi jews whose carriers would benefit from TRID-based therapies. Are there more recurrent nonsense mutations in USH genes?

Response to comment 2: Following reviewer´s suggestions we have included on page 5 of the

introduction the relative frequency of in-frame nonsense mutations in other USH genes (see also response to comment 1):

> *... in-frame nonsense mutations represent ~ 20% of all identified different USH causing mutations (https://grenada.lumc.nl/LOVD2/Usher_montpellier/USHbases. htlm) ...*

The suppression of *PCDH15* nonsense mutations, underlying USH1F were already in focus of previous studies which are cited in our present manuscript (Nudelmann et al. (2006) *Bioorg. Med. Chem. Lett.* **16**, 6310-6315; Rebibo-Sabbah et al. (2007) *Hum. Genet.* **122**, 373-381). In those studies, the designer aminoglycosides showed efficient read-through activity over the standard aminoglycosides. However, several limitations to progress in this project, including the lack of functional assays for the *PCDH15* gene product, forced us to focus on USH1C for which we had already established necessary tools.

Comment 3) The study takes most concerns about TRIDs into account, using a variety of experiments. In particular, the assessment of the functional properties of rescued harmonin is important to (largely) rule out adversive effects of the random residue that is inserted at the position of the premature stop codon. One concern, however, is the unwanted read-through in pseudogenes that may be reactivated and potentially cause side effects. A possible way to address this would be an assay that looks at the read-through of genes that have been inactivated very recently in human evolution and that are still largely intact apart from recently gained nonsense mutations. I think it would be beyond the scope of this study to add such experiments for a revision, but I think that this issue needs to be discussed at least.

Response to comment 3: We agree with the reviewer. We followed her/his suggestions and additionally discussed the issue of off-target read-through of pseudogene activation on pages 11 and 13, respectively:

> *"The read-through therapy might have an inherent problem due to potential off-target effects, e. g. on normal protein translation processes or the reactivation of pseudogenes, inducing side effects. Thus, the biocompatibility of TRIDs in different tissues and in the organism is an important concern (Linde et al, 2008).*"

> *"All in all, we did not find any indication for harmful prolonged protein translation or reactivation of evolutionary turned-off pseudogenes induced by TRIDs mediated read-through of normal termination codons. Thereby, our data further support the hypothesis that normal and premature termination differ mechanistically (Welch et al, 2007)."*

Comment 4) Would the co-administration of NB54 and PTC124 be beneficial in terms of enhanced recovery, as an alternative or in addition to co-administered PAA (which has been shown to be low)? A very good point in this study is that besides pure quantification of recovered FL protein, the functional recovery has been assayed.

Response to comment 4: We thank the reviewer for the comment. We followed the reviewer´s suggestion and performed co-administrations of PTC124 and NB54. We did not observe any synergistic effects.

We describe the results of our co-administration experiments on page 7 and have added Fig. S1 of Supporting Information for the illustration of these results in the Supporting Information:

"Pharmacokinetic and pharmacodiamic performance indicate that NB54 and PTC124 target the translation machinery in different ways (Finkel, 2010), consequently a synergistic effect of combined TRIDs application is reasonable. We evaluated the read-through efficiency following co-administration of NB54 and PTC124. As described above, we transfected HEK293T cells with mutated cDNA coding for harmonin a1, added NB54, or PTC124 or a combination of both drugs to the cells, and compared recovered full-length harmonin a1 expression in Western blot analyses (Fig. S1A of Supporting Information). We normalized the band intensity of recovered hamonin to the band intensity of actin. The quantification of the normalized band intensities revealed no significant increase of harmonin expression following co-administration of NB54 or PTC124 compared to single treatments (Fig. S1B of Supporting Information) indicating no synergistic effect induced by the co-administration of both TRIDs."

Fig. S1 of Supporting Information

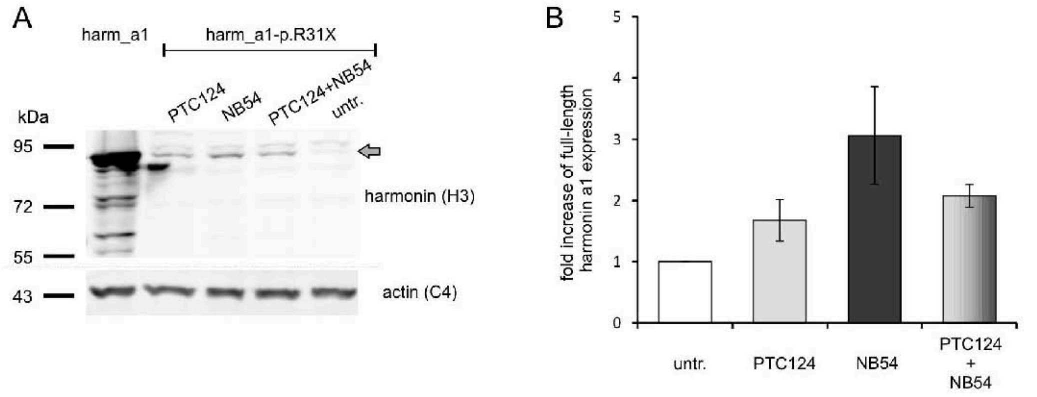

The legend for supplementary Fig. S1 will be found on page 32.

"Figure S1 of Supporting Information. Western blot analyses of read-through induced by TRIDs co-administration. *(A) Read-through in transient harm_a1-p.R31X transfected HEK293T cells following single and co-administration of NB54 and PTC124 analyzed by Western blots with anti-harmonin (H3) and anti-actin (C4) as loading control. Single and co-administration of NB54 or PTC124 restored full-length harmonin a1 (~ 80 kDa) in p.R31X transfected cells. In the 1st lane the expression of harmonin a1 in wild type harm_a1 transfected HEK293 cells is shown. (B) Quantification of TRID mediated read-through of the p.R31X mutation. For the quantification optical densities of harmonin a1 bands were ascertained and normalized to the corresponding loading control. The increase of read-through is shown as fold increase over untreated (untr.) cells. Quantification revealed no synergistic effect induced by co-administration of NB54 and PTC124. Quantitative data resulted from three to five independent repeats of the experiments. Error bars represent SD."*

In addition we added a brief methodical description in the Material and Methods part on page 16:

"For evaluating the potential of a synergistic activity of NB54 and PTC124; 0.5 mg/ml NB54 and 5 µg PTC124, in single or in co-administration were used."

Response to Referee #2:

Reviewer 2 pointed out that our results are supported by solid and well-controlled experiments. She/he criticizes that we are a bit overenthusiastic in describing some of the results.

We see the point and restrained our enthusiasm in describing some of the results:

On page 15 in the sentence "Here we demonstrate the enormous potential of NB54 and PTC124 … we deleted "~~enormous~~". On page 20 we changed the sentence ... *with the sustained read-through efficacies* ... to ... *with the good read-through efficacies*.

Comment 1) The authors describe the read-through efficacy in vivo as being sustained, however only a single evaluation of harmonin a1 was examined 72 hours post treatment. Later time points would need to be evaluated to support concluding a sustained effect, otherwise the effect should be described as transient.

Response to comment 1: We agree with the reviewer that "sustained" in this context might be semantically misleading. We change the sentence to (page 20):

"*The high biocompatibilities combined with the good read-through efficacies of these drugs emphasize the potential of NB54 and PTC124 in treating nonsense mutation-based retinal disorders."*

Comment 2) The authors state that all three harmonin subclasses are expressed in the retina, yet only one subclass, a1, was evaluated in vivo. An analysis of harmonin b would greatly strengthen

the conclusion of in vivo efficacy.

Response to comment 2: In the present manuscript we focused in the *in vivo* analyses on the hamonin a1 isoform since the expression level of this isoform is the most abundant in the retina.

Response to Referee #3:

Comment 1) It is more valuable if the author could include the comparison between NB30, NB54, PTC124 and NB84.

Response to comment 1: We agree with the reviewer, but in our opinion the inclusion of additional designer aminoglycosides goes beyond the scope of the present study. The set-up of all the *in vitro* and *in vivo* experiments is highly time-consuming. Although we are currently investigating other most recent TRIDs for further improvement of efficacy, these data will be reported in due course.

Comment 2) In page#4 on the line#18, The author should describe that PTC proceeded to phase#3 for clinical trial for DMD patients although it has been suspended.

Response to comment 2: We followed the reviewer´s suggestion and included the following sentence on page 4 of the revised manuscript:

> "*With respect to DMD, a randomized, double-blind, placebo-controlled phase IIb trial was carried out. Application of PTC124 was safe over a 48 week treatment period; however the ambitious primary endpoint did not reach statistical significance (http://ptct.client.shareholder.com/releasedetail.cfm?ReleaseID= 518941). Currently a detailed subgroup analysis of the trial is on-going.*"

Comment 3) The data shown in figs. 2B, 4B and 7A, the author should indicate the level of wild type animals. Otherwise, readers cannot understand the significance of the data clearly.

Response to comment 3: The purpose of our experiments illustrated in Figs. 2B, 4B and 7A was to determine the translation read-through efficiencies of TRIDs for the nonsense mutation p.R31X in *USH1C*. For this we present in all three figures the control untreated mutation p.R31X as reference. For the assay in the cell culture, we calculated the percentage of translational read-through in p.R31X-transfected TRID treated cells to wild type harm_a1 transfected cells which we present on page 7 and in Table 1 of our manuscript. We achieved 3.7% recovered full-length harmonin expression with NB54, 2.1% for NB30 and 2.5% for PTC124. Our obtained recovery levels are in line with previously published data (e.g. Keeling et al., 2002), which are in the range to slow down the progression of the disease (Kellermayer, 2006; Maire, 2001).

To highlight the quantification we added the half sentence "*To estimate the relevance of the rescued harmonin expression, ...*" to the description of this calculation on page 6:

> "*To estimate the relevance of the rescued harmonin expression, we calculated the percentage of restored harmonin protein as the ratio of harmonin expression in p.R31X-transfected TRID treated cells, to that of cells transfected with wildtype harmonin lacking the p.R31X mutation. We achieved the highest amount of recovered total harmonin expression with NB54, which yielded in a 3.7% recovery of total harmonin expression compared to 2.1% and 2.5% for NB30 and PTC124, respectively (Table 1). In summary, all TRIDs were able to rescue translational read-though of the p.R31X mutation to some degree resulting in full-length harmonin a1 with the highest level of read-through achieved by NB54.*"

Comment 4) In page#16, it may be possible that the author used not one-way t-test but one-way ANOVA.

Response to comment 4: As indicated in the Materials and Methods (page 18) we used the one-way t-test in our analyses.

2nd Editorial Decision 16 July 2012

Thank you for the submission of your revised manuscript to EMBO Molecular Medicine. We have now received the final reports from the referees that were asked to re-assess it and they have indicated their satisfaction with the revisions.

I am pleased to inform you that we will be able to accept your manuscript pending the following final amendments:

-Please revise all figures to increase label sizes (as the figures may be reduced in the pdf format). When a western blot is shown, please mark the edges with a black line, reduce the contrast settings and do not crop too close from the bands of interest. Make sure to indicate clearly on a figure if two blots were put together to mimic a one-WB experiment. Please make also sure that the figures remain at high quality (see below).

-Could you please provide a Table of Content for the supplementary infrmation even though only 1 file is provided.

-Please split your final article from the point-by-point responses at the beginning and the supplementary figure legend at the end. Remove any yellow highlighting. Please proof-read your article one more time without changing any meanings to the text.

Please submit your revised manuscript within two weeks. I look forward to seeing a revised form of your manuscript as soon as possible.

Yours sincerely,

Editor
EMBO Molecular Medicine

***** Reviewer's comments *****

Referee #1:

All concerns have been sufficiently addressed.

2nd Revision - Authors' Response 17 August 2012

(All requested changes have been made)
