# Supplementary material for: A comparative evaluation of NB30, NB54 and PTC124 in translational read-through efficacy for treatment of an USH1C nonsense mutation
Source: EMBO Mol Med. 2012 Oct 2;4(11):1186–99. doi: 10.1002/emmm.201201438 (PMC3494875; doi:10.1002/emmm.201201438)
Supplement: Supplementary file 2 [file emmm0004-1186-SD2.pdf]

Table of contents.

1. Supplement figure 1. Western blot analyses of read-through induced by TRIDs co-administration.

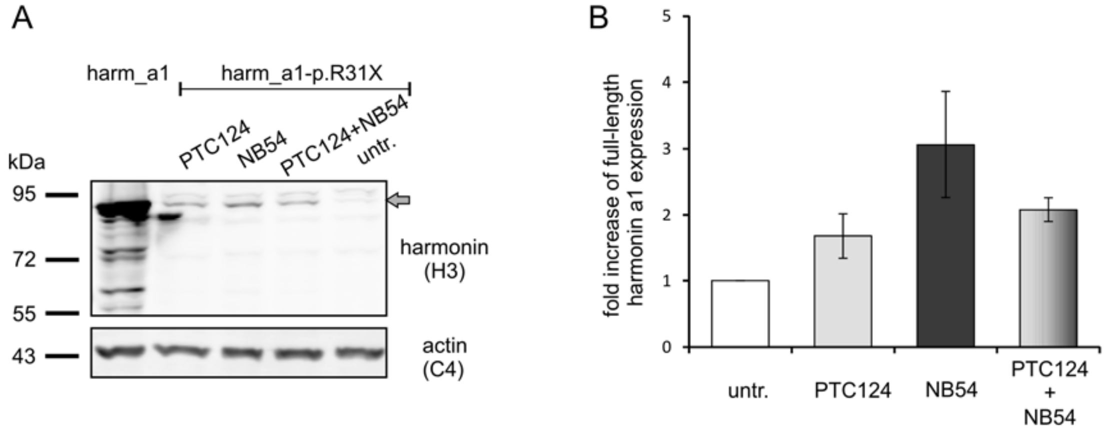

Fig. S1 Western blot analyses of read-through induced by TRIDs co-administration.
(A) Read-through in transient harm_a1-p.R31X transfected HEK293T cells following single and co-administration of NB54 and PTC124 analyzed by Western blots with anti-harmonin (H3) and anti-actin (C4) as loading control. Single and co-administration of NB54 or PTC124 restored full-length harmonin a1 (~ 80 kDa) in p.R31X transfected cells. In the 1st lane the expression of harmonin a1 in wild type harm_a1 transfected HEK293 cells is shown. **(B)** Quantification of TRID mediated read-through of the p.R31X mutation. For the quantification optical densities of harmonin a1 bands were ascertained and normalized to the corresponding loading control. The increase of read-through is shown as fold increase over untreated (untr.) cells. Quantification revealed no synergistic effect induced by co-administration of NB54 and PTC124. Quantitative data resulted from three to five independent repeats of the experiments. Error bars represent SD.
